# Addressing Stroke Care: Overcoming Contrast-Allergy Challenges With Rapid Intravenous Dye Preparation

**DOI:** 10.7759/cureus.90098

**Published:** 2025-08-14

**Authors:** Karen Greenberg, Jennifer A Ross, Thomas A Kurtz, Abby E Deans, Erol Veznedaroglu, Kenneth Liebman, Zakaria Hakma, Rudy Rahme, Mandy Binning

**Affiliations:** 1 Neurosciences, Global Neurosciences Institute, Pennington, USA; 2 Radiology, Main Line Health, Bryn Mawr, USA

**Keywords:** acute ischemic stroke, computed tomography angiography (cta), emergent neuroimaging protocols, large vessel occlusion detection, radiocontrast media allergy

## Abstract

Background: Hypersensitivity reactions to iodinated contrast pose a challenge when urgent neuroimaging is needed in acute stroke. Limited protocols exist for high-risk patients in emergent settings.

Purpose: To evaluate the safety and efficacy of an emergent intravenous (IV) dye preparation protocol for stroke-alert patients with known contrast allergies, enabling timely neuroimaging.

Methodology: This retrospective study reviewed stroke-alert patients from 2021 to 2023 at a comprehensive stroke center. Patients with documented IV dye allergies but no history of airway edema or anaphylaxis were included. An emergent IV dye preparation protocol was administered, consisting of diphenhydramine 50 mg IV, famotidine 20 mg IV, and dexamethasone 10 mg IV before contrast CT angiography (CTA) or CT perfusion (CTP). Data analysis included descriptive statistics, and statistical significance was determined using chi-square tests, with a *P*-value threshold of 0.05.

Results: Of 1,001 stroke-alert patients, 58 (5.8%) had a contrast allergy. Twenty-five of these (43%) received emergent contrast imaging; 16 (64%) were female, with a mean age of 66 ± 15 years. Six of 25 (24%) had a large vessel occlusion or severe carotid stenosis detected by CTA/CTP, compared to two of 26 (7.7%) in the non-contrast group (attributable risk 0.16; 95% confidence interval (CI) -0.07 to 0.39; *P *= 0.11). No adverse reactions occurred within 24 hours post-imaging. The number needed to treat was six.

Conclusions: The emergent IV dye preparation protocol was safe, enabled timely imaging, and improved detection of treatable large-vessel occlusions in acute stroke patients with contrast allergies.

## Introduction

Stroke is the fifth leading cause of death in the United States and a significant contributor to chronic disability and mortality globally [1]. Since its inception in the late 1960s, computed tomography (CT) has transformed diagnostic imaging, facilitating the rapid and accurate assessment of conditions like stroke. In the United States alone, more than 80 million CT scans are now performed annually, compared with just 3 million in 1980 [2]. While CT technology has evolved over time, enabling lower doses of contrast per patient, the rise in CT scans and enhanced imaging procedures has driven a 250% increase in the contrast media market from 2010 to 2018 [2]. Classic stroke alert protocols prioritize timely neuroimaging, including CT angiography (CTA) and CT perfusion (CTP) in the Emergency Department (ED), which are crucial for diagnosing acute ischemic strokes (AIS) and identifying large vessel occlusions (LVO). However, challenges arise when patients with documented allergies to iodinated contrast require emergent imaging.

Recent statistics on the incidence of allergies to radiocontrast media (RCM) indicate that the prevalence remains significant, though estimates vary. Reported incidence ranges from 1,428/196,081 (0.73%) to 2,000/100,000 (2%), depending on the agent and population studied [3]. This includes both immediate reactions within one hour and non-immediate reactions occurring hours to days later. Over 80 million clinical CT scans are performed yearly in the United States alone, which equates to 350,000 to 1.4 million hypersensitivity reactions per year [4].

In the context of stroke patients requiring emergent neuroimaging, the risk of anaphylaxis related to iodinated contrast dye is a significant concern, particularly given the potential for severe outcomes and the associated legal implications. An analysis of anaphylaxis-related malpractice lawsuits highlights that intravenous (IV) contrast was the most common trigger, implicated in 40% of cases. Notably, nearly half of the lawsuits stemmed from exposure to a known allergen, underscoring the importance of stringent safeguards and protocols in preventing such incidents. The legal ramifications of these cases are significant, with a substantial proportion resulting in findings of negligence or settlements [5]. Given these findings, it is imperative to develop protocols for safely administering contrast media in high-risk patients, such as those with a known history of contrast dye allergy, to mitigate both patient harm and legal risk.

The American College of Radiology (ACR) advocates for the use of premedication to mitigate the risk of adverse reactions in patients with a known history of allergic reactions to contrast media. The rationale for premedication is based on reducing the likelihood of both mild and severe reactions [6]. Premedication generally involves the administration of corticosteroids and antihistamines. Corticosteroids are used for their anti-inflammatory properties, which help reduce the body's hypersensitivity response. Antihistamines are used to counteract histamine release, which contributes to allergic symptoms such as urticaria, angioedema, and respiratory distress [6].

The ACR provides detailed recommendations and protocols for elective premedication. These guidelines include the administration of oral steroid medications 12 to 13 hours before imaging. Additionally, protocols for accelerated IV premedication are specified, which involve administering corticosteroids and diphenhydramine four to five hours before imaging. It is important to note that there is no documented evidence available to support the efficacy of premedication durations of two hours or less, regardless of whether the medications are administered orally or intravenously, and whether they are corticosteroid or antihistamine-based [6].

Given the lack of well-established, evidence-based protocols for emergent preparation, we present our institution's practice for patients with documented iodinated IV contrast allergy, excluding those with a history of airway edema or anaphylaxis. Unlike existing accelerated premedication guidelines, which typically require several hours for administration before imaging, our protocol is designed for immediate use at the time of patient presentation, enabling contrast-enhanced neuroimaging within minutes. Our specific objective is to evaluate the effectiveness of this emergent IV dye preparation protocol in expediting advanced neuroimaging without adverse outcomes. While our protocol is focused on stroke patients, it offers a potential framework for expanding emergent preparation to other life-threatening conditions such as pulmonary embolism, aortic dissection, and trauma.

## Materials and methods

Study design and registration

This retrospective case study was conducted at a single center and registered with the research registry under the number NCT05905900. Institutional Review Board (IRB) approval was obtained to perform a data collection via a chart review/retrospective study.

Emergent IV dye preparation protocol

An iodinated IV dye preparation protocol was developed for emergent imaging, involving the administration of diphenhydramine 50 mg IV, Famotidine 20 mg IV, and Dexamethasone 10 mg IV. This preparation was administered immediately before CTA and/or CTP. The contrast agent used was Iodixanol (Visipaque) Injection, an iodinated iso-osmolar, isotonic contrast agent. Patients received 40 mL of contrast for CTP and 90 mL for CTA head and neck, totaling 130 mL.

Study population and setting

The protocol was performed at a busy tertiary teaching hospital and comprehensive stroke center. The study period, from 2021 to 2023, was chosen to coincide with the implementation of a new electronic health system, Cerner, which facilitated data capture. The study focused on stroke-alert patients requiring advanced neuroimaging who had documented iodinated IV dye allergies. Patients with a history of airway edema or anaphylaxis related to contrast dye were excluded from the study. CT technicians, along with emergency physicians, observed patients for adverse events after receiving the emergent iodinated IV dye preparation protocol. Observers were not blinded to group allocation, as direct awareness of protocol administration was essential for appropriate and timely monitoring of potential contrast-related reactions or complications.

The final study cohort was divided into two groups. The first group consisted of 25 patients with a documented IV dye allergy who received the emergent preparation protocol, followed by iodinated contrast administration and CT imaging with contrast (Table 1). The second group included 26 patients with a known contrast media (radiographic contrast media (RCM)) allergy who did not receive the emergent preparation and, as a result, underwent neuroimaging with only a non-contrast head CT (Table 2).

**Table 1:** Summary of patients who received emergent IV dye preparation before advanced neuroimaging. PCN, Penicillin; ASA, Aspirin; ACEI, angiotensin-converting enzyme inhibitor; CT, computed tomography; CTP, CT perfusion; CTA, CT angiography

Age	Gender	Allergies	Reaction	Adverse outcome	Type of media	Image modality/volume
52	Female	IV dye, Shellfish	Hives	None	Iodixanol	CTP/CTA head and neck, 40 mL/90 mL (130 mL total)
69	Female	IV dye	Vomiting/hives	None	Iodixanol	CTP/CTA head and neck, 40 mL/90 mL (130 mL total)
60	Female	IV dye	Unknown	None	Iodixanol	CTP/CTA head and neck, 40 mL/90 mL (130 mL total)
60	Male	IV dye	Hives	None	Iodixanol	CTP/CTA head and neck, 40 mL/90 mL (130 mL total)
50	Male	IV dye, Shellfish	Sob	None	Iodixanol	CTP/CTA head and neck, 40 mL/90 mL (130 mL total)
66	Female	IV dye	Unknown	None	Iodixanol	CTP/CTA head and neck, 40 mL/90 mL (130 mL total)
69	Male	IV dye, Levaquin, PCN	Sob	None	Iodixanol	CTP/CTA head and neck, 40 mL/90 mL (130 mL total)
73	Female	IV dye, ACEI, PCN, ASA	Itching/rash	None	Iodixanol	CTP/CTA head and neck, 40 mL/90 mL (130 mL total)
52	Female	IV dye, Aspartame, ASA, Plavix, PCN, Pork, Prednisone, Shellfish	Hives	None	Iodixanol	CTP/CTA head and neck, 40 mL/90 mL (130 mL total)
58	Male	IV dye, Flagyl, Shellfish, Iodine, Metoprolol, PCN	Rash	None	Iodixanol	CTP/CTA head and neck, 40 mL/90 mL (130 mL total)
68	Female	IV dye, Cefadroxil, PCN, Alirocumab, Latex, statins	Hives	None	Iodixanol	CTP/CTA head and neck, 40 mL/90 mL (130 mL total)
49	Female	IV dye	Rash	None	Iodixanol	CTP/CTA head and neck, 40 mL/90 mL (130 mL total)
40	Male	IV dye, Shellfish, Sulfa	Swelling of the lower extremities	None	Iodixanol	CTP/CTA head and neck, 40 mL/90 mL (130 mL total)
75	Male	IV dye	Rash	None	Iodixanol	CTP/CTA head and neck, 40 mL/90 mL (130 mL total)
70	Male	IV dye, Shellfish	Rash	None	Iodixanol	CTP/CTA head and neck, 40 mL/90 mL (130 mL total)
61	Female	IV dye	Shortness of breath	None	Iodixanol	CTP/CTA head and neck, 40 mL/90 mL (130 mL total)
63	Female	IV dye	Unknown	None	Iodixanol	CTP/CTA head and neck, 40 mL/90 mL (130 mL total)
79	Female	IV dye	Unknown	None	Iodixanol	CTP/CTA head and neck, 40 mL/90 mL (130 mL total)
63	Female	IV dye	Hives	None	Iodixanol	CTP/CTA head and neck, 40 mL/90 mL (130 mL total)
74	Female	IV dye	Rash	None	Iodixanol	CTP/CTA head and neck, 40 mL/90 mL (130 mL total)
79	Male	IV dye	Shortness of breath	None	Iodixanol	CTP/CTA head and neck, 40 mL/90 mL (130 mL total)
57	Male	IV dye, Seafood, Latex	Hives	None	Iodixanol	CTP/CTA head and neck, 40 mL/90 mL (130 mL total)
93	Female	IV dye	Unknown	None	Iodixanol	CTP/CTA head and neck, 40 mL/90 mL (130 mL total)
82	Female	IV dye	Itching	None	Iodixanol	CTP/CTA head and neck, 40 mL/90 mL (130 mL total)
82	Female	IV dye	Rash	None	Iodixanol	CTP/CTA head and neck, 40 mL/90 mL (130 mL total)

**Table 2:** Characteristics of patients with documented radiocontrast media (RCM) allergy who did not undergo emergent IV dye preparation. Initial neuroimaging was limited to non-contrast head CT, with subsequent MRI/MRA performed in a delayed manner as part of the continued stroke evaluation. PCN, Penicillin; ASA, Aspirin; ACEI, angiotensin-converting enzyme inhibitor; MRA, magnetic resonance imaging; LVO, large vessel occlusion

Age (years)	Gender	Allergies	Reaction	MRA + LVO
75	Female	Ciprofloxacin, IV dye, Lyrica, Pineapple, PCN	Throat closing, hives, swelling, itching	None
62	Female	Iodine, IV dye	Unknown	None
43	Female	ACEI, Lovenox, pineapple, shellfish, peanuts, shrimp	Angioedema, Hives, throat closing	None
82	Male	Cefazolin, Bactrim, IV dye, Macrobid, Gadolinium contrast	Bronchospasm, Hives, Chest tightening	None
78	Female	Iodine, IV dye	Unknown	None
55	Female	Iodine, IV dye	Unknown	None
86	Female	Iodine, IV dye	Unknown	+ Right M1 occlusion
71	Male	Iodine, IV dye, Ibuprofen	Anaphylaxis, angioedema	None
78	Female	IV dye	Unknown	None
91	Female	IV dye, metronidazole, Grapes, Pravastatin, Fentanyl, ASA, APAP	Unknown	None
60	Female	IV dye, Percocet, Levofloxacin, Tramadol, Ketorolac	Itching, vomiting, swelling	None
77	Female	IV dye, Shrimp	Throat swelling	None
33	Female	IV dye, Iodine	Itching	+ Left M1 occlusion
83	Female	IV dye, PCN	Hives	None
42	Female	IV dyes, seafood, shellfish, iodine	Hives	None
57	Male	IV dye, seafood, Latex	Anaphylaxis	None
56	Male	IV dye	Itching, general malaise	None
63	Female	Iodine	Anaphylaxis	None
87	Female	IV dye, PCN, Sulfa	Anaphylaxis, rash	None
60	Female	IV dye	Unknown	None
62	Male	IV dye	Swelling	None
83	Male	Atorvastatin, IV dye	Unknown	None
70	Male	IV dye	Shortness of breath	None
66	Female	Latex, Percocet, IV dye	Itching, nausea	None
75	Female	ASA, Cardizem, Codeine, Morphine, Shellfish, IV dye, Sulfa	Angioedema, anaphylaxis, hives	None

Retrospective data collection

Data were retrospectively collected by a multidisciplinary team, including an emergency physician, a neurosurgeon, a research associate, and a medical student on a research elective. Between November 2021 and October 2023, 1,172 medical records of stroke alerts were identified. Of these, 1,001 records were reviewed for the first inclusion criterion: a documented history of allergies to IV dye, iodinated contrast agents, or iodine.

Data analysis

The primary objective was to assess the association between documented IV dye allergies and potential adverse reactions following the emergent iodinated IV dye preparation. Subsequent results were analyzed to identify any correlation between prior contrast dye allergy and the occurrence of allergic reactions after the emergent iodinated IV dye administration.

Statistical methods

Descriptive statistics were used to summarize patient demographics, allergy history, and the incidence of adverse reactions. Chi-square analysis was used to compare outcomes between the treatment and control groups, with the *P*-value of significance set to 0.05. The results are displayed with 95% confidence intervals (Koopman asymptomatic score method), attributable risk (Newcombe/Wilson method), and the reciprocal of attributable risk, or number needed to treat (NNT). The sample size was determined by the total number of eligible patients meeting inclusion criteria during the study period (2021-2023), as this retrospective analysis included all consecutive stroke-alert patients with documented iodinated IV contrast allergy who met the protocol criteria.

## Results

Descriptive data

From 2021 to 2023, 1,001 stroke-alert patient records were reviewed. Of these, 58 (5.8%) had a documented allergy to iodinated IV contrast. Among these 58 patients, 37 (63%) were female and 21 (37%) were male.

Of the 58 patients with IV contrast allergies, 33 (57%) were considered to have a low suspicion of LVO as defined in our protocol by a National Institutes of Health Stroke Scale (NIHSS) score less than 4 and underwent non-contrast neuroimaging. From this group, 26 patients were selected as matched controls, ranging in age from 33 to 93 years, including 18 (69%) females and 8 (31%) males (Table 3).

**Table 3:** Summary of patient demographics and imaging approach by group. Of the 1,001 stroke-alert patients reviewed, 58 (5.8%) had a documented allergy to iodinated IV contrast. Control and treatment groups were derived from this subset based on imaging pathway and clinical suspicion for large vessel occlusion (LVO). The treatment group underwent contrast-enhanced imaging following emergent IV dye preparation.

Group	N	Female, *n* (%)	Male, *n *(%)
All stroke alerts	1,001		
Contrast allergy	58	37 (63%)	21 (37%)
Control group	26	18 (69%)	8 (31%)
Treatment group	25	16 (64%)	9 (36%)

In this cohort, 2/26 (7.7%) LVOs were detected by MRI/MRA imaging in a delayed fashion after admission as part of continued stroke workup. The most frequently reported allergic reactions included hives, rash, swelling, and shortness of breath.

Results

In the treatment group, 25 patients with known IV contrast allergies underwent neuroimaging with contrast agents following the emergent IV dye preparation protocol. This group ranged in age from 40 to 93 years and included 16 (64%) females and 9 (36%) males. Among these patients, 6 (24%) received Tenecteplase, and 6 (24%) had an LVO or severe carotid stenosis requiring intervention.

Regarding allergy history, 13 of 25 (52%) patients reported a documented allergy to iodinated IV contrast only, while 12 of 25 (48%) had multiple allergies, including iodinated IV contrast. Seven of 25 (28%) patients had unspecified reactions due to aphasia or altered mental status and were empirically treated with the protocol. Reported symptoms included hives in 5 of 25 (20%), rash in 5 of 25 (20%), shortness of breath in 4 of 25 (16%), itching in 2 of 25 (8%), vomiting in 1 of 25 (4%), swelling in 1 of 25 (4%), and unknown in 7 of 25 (28%) (Table 4).

**Table 4:** Documented symptoms among 25 patients with a known iodinated IV contrast allergy who received contrast-enhanced imaging. Seven patients (28%) were unable to specify reaction type due to aphasia or altered mental status and were empirically treated. Hives and rash were the most commonly reported symptoms.

Reaction type	*n* (%)
Hives	5 (20%)
Rash	5 (20%)
Shortness of breath	4 (16%)
Itching	2 (8%)
Vomiting	1 (4%)
Swelling	1 (4%)
Unknown	7 (28%)

None of the 25 patients in the treatment group experienced adverse reactions following neuroimaging with contrast agents. A difference was noted in LVO detection, with 6 of 25 patients (24%) in the treatment group having LVO or severe carotid stenosis identified by CTA/CTP, compared with 2 of 26 patients (7.7%) in the control group, whose cases were detected later by MRI/MRA (Table 5).

**Table 5:** Comparison of outcomes between treatment and control groups. The treatment group demonstrated a higher rate of LVO or severe carotid stenosis detection (24%) compared to delayed detection in the control group (7.7%). No adverse reactions occurred in the treatment group.

Group	Patients with LVO, *n* (%)	Tenecteplase administered, *n* (%)	Adverse reactions, *n* (%)
Treatment group	6 (24%)	6 (24%)	0 (0%)
Control group	2 (7.7%)	-	-

This difference resulted in an attributable risk of 0.16 (95% confidence interval: -0.07 to 0.39, *P* = 0.11). The calculated NNT was 6, meaning that for every six patients receiving the iodinated IV dye preparation, one LVO would be identified by CTA/CTP that would have otherwise gone undetected without the administration of contrast dye. Clinically, this could translate into additional patients receiving timely endovascular thrombectomy (EVT) or other reperfusion therapy, potentially improving functional outcomes and reducing long-term disability.

## Discussion

The treatment landscape of acute ischemic stroke (AIS) is rapidly evolving, with time being a critical determinant of patient outcomes. Early studies highlighted significant neuronal loss in untreated stroke at approximately 1.9 million neurons per minute, and recent findings show higher rates in some patients due to collateral circulation dynamics [7,8]. Our institution's retrospective review identified 6 of 25 (24%) patients with LVO in the treatment group. This percentage aligns with national estimates [9,10].

LVOs pose a significant clinical challenge due to their high morbidity and mortality without intervention. EVT) dramatically improves outcomes for LVO patients, significantly reducing 90-day disability [9,10]. Accurate and timely neuroimaging is crucial for distinguishing LVO from non-LVO strokes, which facilitates prompt intervention.

The ACR provides guidelines for accelerated dye preparation but does not specify protocols for emergent studies where preparation times need to be minimized [6]. While most hospitals have IV dye preparation protocols based on best medical practices, no formal guidelines exist for emergent imaging in patients with contrast allergies.

Our protocol results demonstrated that of the 25 patients who received emergent iodinated IV dye preparation, none experienced adverse outcomes during emergency department evaluation or up to 24 hours after admission. While no immediate or early delayed reactions were observed, the possibility of allergic reactions occurring beyond the 24-hour monitoring period cannot be excluded, and therefore, some delayed events may have gone undetected. The findings support the use of emergent iodinated IV dye preparation for patients with a history of mild to moderate contrast reactions, ensuring timely diagnosis and treatment. Furthermore, this protocol could potentially benefit the diagnosis of other critical conditions requiring advanced imaging, such as pulmonary embolism, aortic dissection, and trauma.

Allergic reactions and anaphylaxis to radiocontrast media (RCM) used in CT scans involve both immunologic and non-immunologic mechanisms. Immunologic mechanisms include IgE-mediated reactions, where IgE antibodies trigger mast cell and basophil degranulation, and T cell-mediated delayed-type hypersensitivity responses [6,11]. Non-immunologic mechanisms involve direct mast cell activation, complement system activation, and effects related to the high osmolality of some contrast agents [11,12]. Clinical manifestations range from mild urticaria to severe, multi-system anaphylaxis. Understanding these mechanisms is crucial for preventing, managing, and diagnosing reactions in susceptible individuals [6,11,12]. Risk factors include previous RCM exposure and older age [10,11]. In one study of 257 patients with a known prior moderate or severe contrast allergy, 87 (33.9%) experienced a recurrent reaction upon re-exposure [13]. This underscores the necessity for implementing a robust pretreatment regimen for identified or suspected patients undergoing emergency imaging studies and procedures.

In managing anaphylaxis, epinephrine remains critical; however, premedication with epinephrine is not considered safe [14]. Therefore, our protocol utilized adjunctive therapies such as diphenhydramine and dexamethasone, recommended by several medical organizations. The World Allergy Organization (WAO), the American Academy of Allergy, Asthma & Immunology (AAAAI), and the European Academy of Allergy and Clinical Immunology (EAACI) all recommend the use of H1 antihistamines and corticosteroids as secondary treatments for symptomatic relief and for the potential reduction of biphasic anaphylaxis risk [15-17].

H2-antagonists, such as famotidine, play a crucial role by blocking the effects of released histamine at H2 receptors, treating vasodilation, some cardiac effects, and glandular hypersecretion. Combining H2 blockers with H1 blockers has an additive benefit over H1 blockers alone in treating anaphylaxis [18]. During the COVID-19 pandemic, our hospital faced a shortage of methylprednisolone and hydrocortisone, making dexamethasone the glucocorticoid of choice. Famotidine was the H2 antagonist of choice, as it was readily available on formulary.

Tonetti et al. reported their experience for 1,521 patients undergoing emergent preparations for IV contrast before mechanical thrombectomy with or without prior CTA. Within that cohort, 43/60 (72%) proceeded directly to endovascular therapy, where patients had a secure airway and contrast reaction would be less worrisome [19]. Our cohort of 25 patients contributes valuable insights to the existing literature, affirming the safety of emergent iodinated IV dye preparation in individuals requiring advanced neuroimaging without the prerequisite of endotracheal intubation.

Jha et al. examined a steroid-only premedication approach for emergent percutaneous coronary intervention in patients with contrast allergies, finding a low incidence of reactions. However, this study was limited by sample size [20]. Our study, involving 25 patients, found no adverse outcomes, suggesting that incorporating histamine receptor antagonists alongside steroids may enhance safety in emergent situations.

This study addresses a critical gap in the management of patients with AIS with documented contrast dye allergies by demonstrating the safety of an emergent IV dye preparation protocol. However, the retrospective design and small sample size limit the generalizability of our findings. The potential for selection bias exists, as patient inclusion was dependent on documented allergy status and clinical decision-making regarding imaging modality. Additionally, the focus on short-term outcomes restricts the assessment of long-term implications in this patient population, and no structured follow-up was conducted beyond the hospitalization period. A notable limitation is that two patients in the control group were later identified to have LVO by MRI/MRA imaging in a delayed fashion. Since most emergency departments in the United States lack access to emergent MRI/MRA, this reliance on delayed neuroimaging underscores the importance of protocols that safely enable timely CTA/CTP in the ED setting.

Further research in larger, multi-center cohorts is necessary to validate these findings and to develop standardized, evidence-based guidelines for managing contrast allergies in emergent settings. Our results support the adoption of an emergent iodinated IV dye preparation protocol, offering a safe and effective strategy for timely neuroimaging critical to AIS management and potentially beneficial for other life-threatening conditions requiring advanced imaging.

## Conclusions

Although most hospitals have an IV dye preparation protocol based on presumed best medical practice, current literature lacks formal guidelines regarding premedication for patients with contrast allergies requiring emergent imaging. Our protocol, involving 25 patients who received emergent iodinated IV preparation, revealed no adverse outcomes during emergency department evaluation and up to 24 hours after admission. The results suggest that a history of mild or moderate contrast reaction should not preclude timely diagnosis and treatment. While broader applicability beyond stroke patients is a potential consideration, including in conditions such as pulmonary embolism, aortic dissection, and trauma, this should be interpreted with caution given the limited sample size and single-center nature of the study. Larger, multi-center studies are needed to confirm these findings.
